# The Effect of Luteinizing Hormone Reducing Agent on Anxiety and Novel Object Recognition Memory in Gonadectomized Rats

**DOI:** 10.18869/nirp.bcn.8.2.113

**Published:** 2017

**Authors:** Paria Arfa-Fatollahkhani, Arezo Nahavandi, Hossein Abtahi, Shabnam Anjidani, Sahar Borhani, Seyed Behnam Jameie, Mohammad Shabani, Saeed Mehrzadi, Ali Shahbazi

**Affiliations:** 1. Department of Neuroscience, School of Advanced Technologies in Medicine, Iran University of Medical Sciences, Tehran, Iran.; 2. Physiology Research Center, Iran University of Medical Sciences, Tehran, Iran.; 3. Department of Physiology, School of Medicine, Iran University of Medical Sciences, Tehran, Iran.; 4. Department of Biochemistry and Nutrition, School of Medicine, Gonabad University of Medical Sciences, Gonabad, Iran.; 5. Department of Biochemistry, School of Medicine, Iran University of Medical Sciences, Tehran, Iran.; 6. Department of Neuroscience, Faculty of Advanced Technologies in Medicine, Iran University of Medical Sciences, Tehran, Iran.; 7. Department of Pharmacology, School of Medicine, Iran University of Medical Sciences, Tehran, Iran.

**Keywords:** Menopause, Andropause, Luteinizing hormone, Gonadectomy, Anxiety, New object recognition memory

## Abstract

**Introduction:**

Mood disorders such as anxiety and depression are common following menopause and andropause. Lack of sex steroid hormones is suggested as the primary cause of these disturbances. The level of luteinizing hormone (LH) would also rise 3–4 times than normal in these people. The potential effects of LH on mood and cognitive symptoms following menopause and andropause are still unknown. This study aimed to investigate the effect of increased LH on novel object discrimination (NOD) memory and anxiety like behavior in gonadectomized rats.

**Methods:**

Four-month-old male and female Wistar rats were randomly assigned into 4 groups (in each sex): control rats (Cont), gonadectomized without treatment (GnX), gonadectomized treated with triptorelin, a GnRH agonist which reduces LH release eventually, (GnX+Tr), gonadectomized treated with triptorelin plus sex steroid hormone, estradiol in female and testosterone in male rats (GnX+Tr+S/T). After 4 weeks treatment, anxiety score (elevated plus maze) and NOD were measured. Data were analyzed using One-way ANOVA, and P-values less than 0.05 were considered as significant.

**Results:**

Gonadectomy increased anxiety like behaviors (decrease of presence time in the open arms) in female rats (P=0.012), but not in male ones (P=0.662). Additionally, triptorelin alone reduced the increased anxiety score in gonadectomized female rats, compared to group treated with both triptorelin and estradiol. Furthermore, it was shown that gonadectomy and or treatment with triptorelin and sex steroids had no significant effect on novel object recognition memory in both female (P=0.472) and male rats (P=0.798).

**Conclusion:**

Findings of this study revealed that increased level of LH following menopause or andropause should be considered as a possible cause for increased anxiety. Also, this study showed that LH reducing agents would reduce anxiety like behavior in gonadectomized female rats. The effect of increased LH on cognitive functions such as novel object recognition memory was not evident in this study and needs further studies.

## 1. Introduction

Cognitive impairments and mood disorders such as memory deficits, anxiety, and depression are common following menopause and andropause (Sherwin 1996; Picazo, Estrada-Camarena et al., 2006). Memory impairments and Alzheimer Disease (AD) are twofold more prevalent in females compared to males, especially in postmenopausal women (Vadakkadath Meethal and Atwood, 2005; Craig and Murphy, 2009). Alterations in hormonal profile of the Hypothalamic–Pituitary–Gonadal (HPG) axis, particularly steroid hormones are one of the main causes of neuropsychiatric disturbances following menopause or andropause (Castanho, Moreira et al., 2014; Blair, McGee et al., 2015).

The neuroprotective and neurogenesis enhancement effects of estrogen on hippocampal neurons have been reported in many studies (Vadakkadath, Meethal, and Atwood, 2005; Craig and Murphy, 2009). As studies suggested, estrogen has many important effects on central nervous system, in addition to its critical effects on reproductive organs. Estrogen can promote memory function and prevent the potential deleterious effects of different neurotoxic agents on hippocampal neurons (Vadakkadath, Meethal, and Atwood, 2005; Craig and Murphy, 2009). It also has been reported that estrogen and progesterone have anxiolytic properties in gonadectomized rats (Walf and Frye, 2006; Frye, Koonce et al., 2008).

Furthermore, several studies have supported neuro-protective and neuromodulatory effects of androgens in cognitive and mood disturbances (Genazzani, Pluchino et al., 2007). It also has been shown that testosterone replacement therapy ameliorated mild cognitive impairments and may postpone the onset of AD in men with andropause (Edinger, Lee et al., 2004). A few studies have revealed that acute injection of testosterone may promote the novel object recognition memory, a cognitive process associated with hippocampus and cortical areas such as perirhinal region (Dere, Huston et al., 2007), in male rats after gonadectomy (Aubele, Kaufman et al., 2008). Another study shows that testosterone promotes the spatial memory in gonadectomized rats, which may be related to the effects of androgenic or estrogenic metabolites of testosterone, or through feedback inhibition of LH release (McConnell, Alla et al., 2012).

Following menopause or andropause, serum level of sex hormones decreases significantly, while the level of Luteinizing Hormone (LH) rises 3–4 times in postmenopausal women and 2–3 times in postandropausal men (Chakravarti, Collins et al., 1976; Vadakkadath, Meethal, and Atwood, 2005). Although not clearly understood, there may be a relationship between cognitive and mood disturbances following menopause and or andropause, and elevated levels of LH. Furthermore, the association of LH with memory impairments and progression of AD has been reported (Vadakkadath, Meethal, and Atwood, 2005; Aubele, Kaufman et al., 2008; Palm, Chang et al., 2014; Blair, Palm et al., 2016). Recently, a cohort study conducted in 2016 demonstrated the effects of reducing LH on the cognitive and spatial memory improvements in the ovarectomized rats (Blair, Palm et al., 2016).

Although the results are not consistent, and sometimes controversial, Hormone Replacement Therapies (HRT) with estrogen alone or in combination with progesterone is a common strategy to reduce the physical and mental complications of menopause (Smith, Giordani et al., 2001; Shumaker, Legault et al., 2003; Low and Anstey, 2006). Methodological differences such as subjects, methods, protocols, and initiation of treatment (immediate or delayed) are proposed as the main factors responsible for various outcomes in HRT clinical trials (Webber, Casadesus et al., 2007). However, the possible deleterious effects of increased LH has not been studied yet.

Little is known about the consequences of elevated levels of blood LH following menopause or andropause, and also the possible beneficial effects of co-administration of steroids with GnRH agonists, which decreases LH release from anterior hypophysis and consequently results in lower level of LH in the blood. This study aims to evaluate the effect of treatment with triptorelin, a sustained release GnRH agonist, which decreases LH release (Asch, Rojas et al., 1985), with or without HRT (β-estradiol in females and testosterone in males) on Novel Object Discrimination (NOD) memory and anxiety in gonadectomized rats.

## 2. Methods

### 2.1. Animals

Eight groups of 4-month-old male and female Wistar rats (n: 9–12 each group) were used in this study. Female rats were assigned into control (Cont), gonadectomized without treatment (GnX), treated with triptorelin (GnX+Tr), and triptorelin plus estradiol (GnX+Tr+S). Male rats were assigned into control (Cont), gonadectomized without treatment (GnX), treated with triptorelin (GnX+Tr), and triptorelin plus testosterone (GnX+Tr+T). All animals were kept in clear Plexiglas cages, 4 in each cage, with free access to food and water ad libitum. Light and dark cycles were 12 hours (lights on at 7:00 AM). The room temperature was kept at 23–24°C. Behavioral tests were carried out between 8:00 and 14:00. Efforts were made to reduce pain and suffering of animals. All the protocols and procedures were in accordance to the roles of Ethics Committee of Iran University of Medical Sciences.

### 2.2. Gonadectomy surgery

Surgical procedures were performed aseptically. In brief, rats were anesthetized with a mixture of ketamine (100 mg/kg, IP) and xylazine (10 mg/kg, IP). Cutting the scrotum about 1.5 cm in male rats, vas deferens were exposed and ligated, and then gonads were resected. In female rats, the ovariectomy procedure was performed with making an incision in the abdominal midline; then the ovaries and the most proximal part of oviduct were removed (Abtahi, et al., 2013). Animals were kept calm and warm after surgery.

### 2.3. Treatment

Treatment with triptorelin alone or with sex hormones was started 10 days after surgery and continued for 4 months thereafter. Triptorelin (Diphereline) was injected intramuscularly 0.5 mg/kg, every 21 days for 4 months; this dose was according to the previous studies that have shown adequate decrease of LH level following this treatment in rats. Female rats, which were treated with estradiol, were injected with estradiol valerate (1.76 mg/ kg, IM) every 14 days for 4 months. Male rats, which were treated with testosterone, received testosterone undecanoate (120 mg/kg, IM) every 8 weeks for 4 months (Abtahi, et al., 2013).

### 2.4. Behavioral tests

Three days before the tests, rats were transferred to the behavioral lab, where they were handled for 5 minutes each day by the examiner. There was at least one day interval between the last injection and behavioral tests. All the behavioral tests were done in the light period of the light/dark cycle. Tests were carried out in a separate room equipped with a remote CCD camera capturing the events, under dim light condition, while controlled by a researcher blinded to the studied groups.

#### 2.4.1. Novel object discrimination test

Rats were put in the open field arena (40×40 cm) for one hour in order to be adapted to the environment. One day later, rats were put in the same arena with two relative heavy solid objects in two opposite corners of the arena for three minutes. Solid objects were different in shape and color (A and B). The time (seconds) spent to explore the objects A and B by the rat was recorded. After 3 minutes, rats were exited from the arena for 1 hour. Then rats returned to the arena and allowed to explore the objects for 3 minutes; while one of the objects (B) was replaced by a new object (C), with different shape and color). The time rats spent to explore each familiar (A) and new object (C) were recorded. Rats, which explored each of the objects for less than 1 second, were excluded from the study. The percentage of the time exploring new object in last test was calculated as the percentage of NOD time. After each test the arena and solid objects were cleaned with ethylic alcohol.

#### 2.4.2. Elevated plus maze test

Elevated Plus Maze (EPM) is a plus shaped apparatus with two open and two closed arms (10 cm wide and 60 cm length) crossed each other, with a 10×10 square area in the crossed central zone. The arms were 50 cm higher than ground. Closed arms have edges with 50 cm height, and open arms with no edge. The day after NOD test, anxiety test (EPM) was carried out. Rats were put in the central zone of the maze, with head toward open arms; and permitted to explore the arms for 5 minutes. The total time of presence in both open (OAT) and closed arms (CAT) were measured. Decreased presence time in open arms (OAT) was considered as anxiety score. Because the lack of difference in presence time in closed arms and also the total number of entries to open and closed arms, these data were not presented here. The total number of entries to both arms are measured and compared, however, no differences were detected between groups (data not shown).

### 2.5. Statistical analyses

SPSS version 16 was used for analysis of data. Oneway ANOVA test was used for comparison of means between groups; when P-value was significant, post hoc Tukey test would be performed. P-values less than 0.05 were considered as significant.

## 3. Results

### 3.1. The effect of treatment with triptorelin alone or with estradiol on anxiety like behavior in female gonadectomized rats

**Figure 1.**
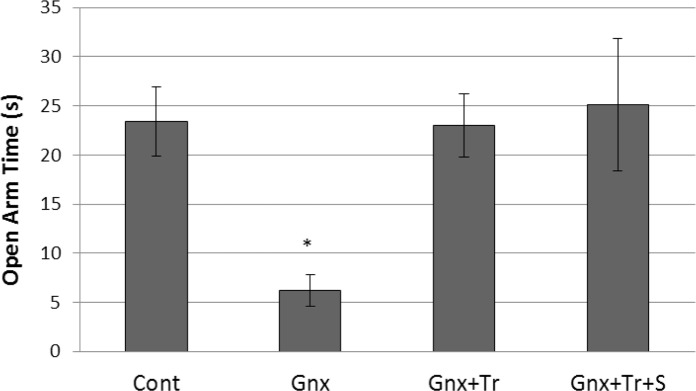
Triptorelin with or without hormone therapy normalized anxiety-like behavior in gonadectomized female rats. Figure 1 presents the mean (SD) of presence time in the open arms (OAT) in female rats (n: 9–12 in each group); including control (Cont), gonadectomized without treatment (GnX), or treated with triptorelin (GnX+Tr), and triptorelin plus estradiol (GnX+Tr+S) (*P<0.05).

Results of 1-way analysis of variance (ANOVA) for comparison the mean of presence time in the open arms (OAT) in female rats showed a significant effect of treatment on OAT [F(3, 34)=4.32, P=0.012]. Post hoc Tukey test revealed that OAT in gonadectomized group (GnX, 6.2±1.6) was significantly lower than the control group (Cont, 23.3±3.5, P<0.05); While, OAT in gonadectomized group treated with triptorelin (GnX+Tr, 23±3.3) and gonadectomized group treated with triptorelin plus estradiol (GnX+Tr+S, 25.1±6.8) were not different from the control group (P>0.05). However, the OAT in gonadectomized groups treated with triptorelin (GnX+Tr) or triptorelin plus estradiol (GnX+Tr+S) was not significantly different (Figure 1).

### 3.2. The effect of treatment with triptorelin alone or with testosterone on anxiety like behavior in male gonadectomized rats

**Figure 2.**
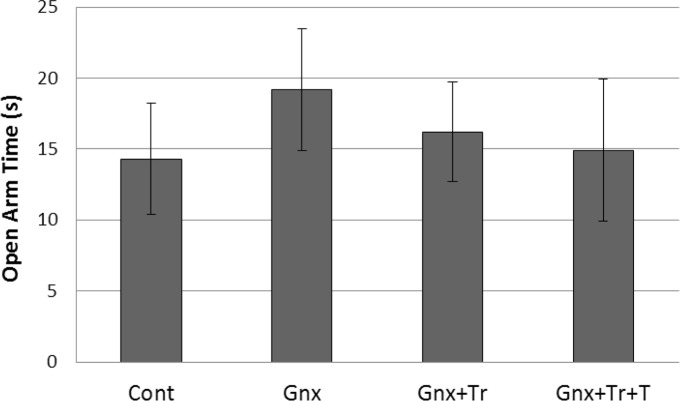
Gonadectomy with or without treatment with triptorelin and or testosterone had no effect on the anxiety like behavior in male rats. Figure 2 presents the mean (SD) of presence time in the open arms (OAT) in male rats (n: 9–12 in each group), including control (Cont), gonadectomized without treatment (GnX), or treated with triptorelin (GnX+Tr), and triptorelin plus testosterone (GnX+Tr+T).

In male rats, the results of 1-way ANOVA for comparing the mean presence time in the open arms (OAT) showed no significant effect of treatment on OAT [F(3, 45)=0.532, P=0.662] in control (Cont), gonadectomized without treatment (GnX), or treated with triptorelin (GnX+Tr), and triptorelin plus testosterone (GnX+Tr+T) groups (Figure 2).

### 3.3. The effect of treatment with triptorelin alone or with estradiol on the novel object discrimination memory in female gonadectomized rats

**Figure 3.**
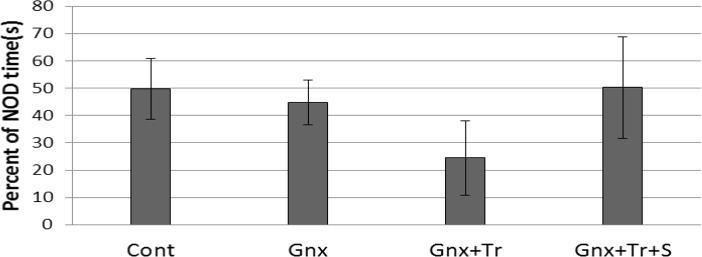
Gonadectomy with or without hormone therapy did not affect novel object discrimination task in female rats.

One-way ANOVA results revealed that the percentage of time exploring the novel object was not significantly different in control (Cont), gonadectomized without treatment (GnX), or treated with triptorelin (GnX+Tr), and triptorelin plus estradiol (GnX+Tr+S) groups [F(3, 27)=0.864, P=0.472] (Figure 3). Figure 3 illustrates comparison of the mean (SD) of exploration time for novel object (NOD) in different female groups (n: 6–9 rats in each group); including control (Cont), gonadectomized without treatment (GnX) or treated with triptorelin (GnX+Tr), and triptorelin plus estradiol (GnX+Tr+S). There was no significant difference between groups.

### 3.4. The effect of treatment with triptorelin alone or with estradiol on the novel object discrimination memory in male gonadectomized rats

**Figure 4.**
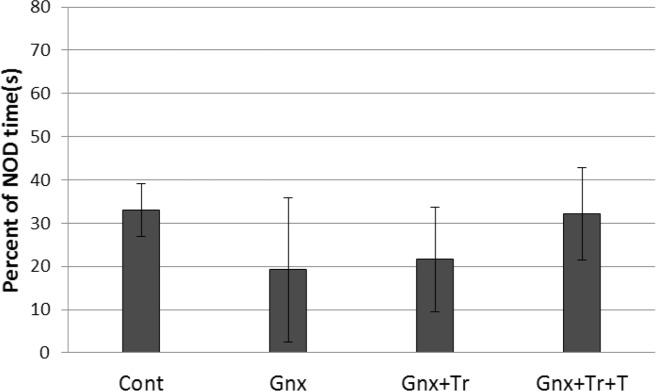
Gonadectomy with or without hormone therapy had no effect on novel object discrimination task in male rats.

Comparing the percentage of time exploring novel object in different male groups (n: 7–9 rats in each group); Control (Cont), gonadectomized without treatment (GnX) or treated with triptorelin (GnX+Tr), and triptorelin plus testosterone (GnX+Tr+T) using 1-way ANOVA did not show any significant difference between groups [F(3, 24)=0. 337, P=0.798] (Figure 4).

Figure 4 presents mean (SD) of exploration time of novel object (NOD) in different male groups (n: 6–9 in each group); Control (Cont), Gonadectomized without treatment (GnX) or treated with triptorelin (GnX+Tr), and triptorelin plus testosterone (GnX+Tr+T). There was no significant difference between groups.

## 4. Discussion

The result of this study demonstrated that gonadectomy has different and gender-specific effects on the anxiety-like behavior in adult rats, which was measured using elevated plus maze test. Here, it was shown that gonadectomy increased the anxiety score in female rats, while it had no effects on anxiety score in male rats. Furthermore, long-term treatment with GnRH agonist, triptorelin (Diphereline), diminished the increased anxiety score in gonadectomized female rats, which was comparable to the effect of combination therapy with triptorelin and estradiol. Additionally, it was shown that gonadectomy and or treatment with triptorelin and sex steroids had no significant effect on the new object recognition memory in both female and male rats.

The higher prevalence of mood disorders in elderly people beyond the age of fertility has been reported in many studies. Anxiety and depression are among the most reported disorders with varying prevalence rates after menopause or andropause (Sherwin, 1996). This study demonstrated that gonadectomy can induce anxiety in female rats, which is in agreement with the reports of higher incidence of anxiety in menopause women (Sherwin, 1996). However, the anxiety score (presence time in open arm) in male gonadectomized rats was not different from the non-gonadectomized control rats, which was in accordance to other studies (Zimmerberg and Farley, 1993). Regarding the deficiency of sex steroid hormones following menopause, or after gonadectomy in animal models, it has been widely accepted that insufficient level of estrogen in menopause women or estradiol in female gonadectomized rats may be responsible for observed higher anxiety in these conditions.

Modulatory functions of estrogen on some signaling process of different neurotransmitters, can potentially affect the anxiety symptoms, i.e., the modulatory role of estrogen on serotoninergic system (Pandaranandaka, Poonyachoti et al., 2009). Additionally, estrogen can also modify some neural functions in hippocampus and amygdala, which have a pivotal role in the regulation of stress response system, via the E2 receptors (Walf and Frye, 2006). This steroid hormone also may exert some effects on the growth and development of hippocampal cells, which can also affect the stress response system. Furthermore, It has been reported that hippocampal excitatory output via the ventral subiculum can modulate the activity of dopaminergic neurons in the ventral tegmentum, and consequently release of dopamine in nucleus accumbens in rodents (Lodge and Grace, 2006), which may related to reward processing and motivated behavior. Therefore, it seems that estrogen can decrease anxiety via different mechanisms (Osterlund, Witt et al., 2005).

However, serum level of LH rises dramatically after menopause because of the lack of sex steroids negative feedback. To our knowledge, there was no study on the potential role of increased level of LH on the anxiety symptoms following menopause. The findings of this study demonstrate the beneficial effects of long-term treatment with GnRH agonist alone, which decrease LH in long term, on the increased anxiety score in female gonadectomized rats. Interestingly, this effect did not improve significantly after addition of estradiol to triptorelin in the group treated with both of them. This finding may be important with regard to the treatment of behavioral and somatic problems in menopause patients, where the only treatment option for reducing the mood and cognitive symptoms is hormone replacing therapy with sex steroids (Low and Anstey, 2006).

The results of this study suggest that the elevated level of LH in these individuals may have devastating consequences and reducing LH may be considered as a potential therapeutic option in these patients. However, the findings of the present study are preliminary, and more studies on the role of elevated LH on the anxiety following menopause is needed. One of the limitations of this study is using just one method to assess anxiety-like behavior, which need to replicate in another anxiety evaluation tests.

We have shown no significant effect of gonadectomy on the novel object recognition memory (NOD) of both male and female rats. However, this finding is not in accordance to the findings of another study (Aubele, Kaufman et al., 2008). Large standard deviation of our data may be the cause of these different results, which was partly due to the exclusion of some rats in some study groups based on the behavioral exclusion criteria. NOD is one of the reliable tests for investigating the function of perirhinal and parahippocampal areas (Aubele, Kaufman et al., 2008). Nevertheless, similar to other memory systems, normal function of hippocampus and prefrontal cortex is also necessary in NOD. High level of LH may affect other memory systems. Some studies have proposed the deleterious effect of LH on senile memory disturbances, such as Alzheimer disease, and suggested that LH may potentiate the pathological mechanisms of these disorders (Meethal, Smith et al., 2005; Casadesus, Milliken et al., 2007; Webber, Casadesus et al., 2007; Craig and Murphy, 2009; Palm, Chang et al., 2014; Blair, Palm et al., 2016).

In conclusion, this study revealed that increased level of LH following menopause should be considered a possible cause of increased anxiety in women. Here, it was shown that long-term treatment with LH reducing agents such as triptorelin could relieve anxiety-like behavior in gonadectomized female rats, which was similar to the treatment with combination of triptorelin and estradiol. It was suggested that addition of LH reducing agents such as sustained GnRH agonists to hormone replacement therapy may have beneficial effects on mood disturbances following menopause; however, more studies are needed. The effect of increased LH on cognitive functions such as new object recognition memory was not shown in this study that should be interpreted with caution and need further studies.
